# Research on issues in the protection of clinical trial human subjects in China: a Delphi study

**DOI:** 10.1186/s12910-025-01302-5

**Published:** 2025-11-14

**Authors:** Xiuqiao Yang, Hong Lin, Shuning Liu, Chunyan Ai, Zhanghong Cai, Rong Yu, Xiaoyi Xu, Jinghong Gao, Yuwen Chen, Ping Wen

**Affiliations:** 1 Department of Clinical Trail Management, Shenzhen Maternity and Child Healthcare Hospital, Women and Children’s Medical Center, Shenzhen, 518028 China; 2 School of Business Administration, Shenyang Pharmaceutical University, Shenyang, Liaoning 110016 China

**Keywords:** Clinical trial, Subject rights, Compensation rights, Privacy rights, Informed consent rights

## Abstract

**Background:**

This study aims to identify current deficiencies in the protection of human subjects participating in clinical trials in China and propose strategies for safeguarding their rights.

**Methods:**

We conducted a comprehensive literature review on the protection of human subjects in clinical trials and gathered insights from experts and scholars among stakeholders. The Delphi expert consultation method was used to analyze and summarize the shortcomings in China’s regulatory framework for human subject protection and to develop strategies for improvement. Two rounds of surveys were conducted with experts from research institutions, ethics committees, regulatory agencies, sponsors, and Site Management Organizations (SMOs). The top three issues and proposed solutions were discussed and addressed based on expert consensus.

**Results:**

A total of 30 articles were reviewed, leading to the identification of 46 distinct issues, which were categorized into three groups: compensation, privacy, and informed consent. Sixteen respondents completed both rounds of the survey, which included 56 questions (10 new questions were proposed in the first round). Compensation-related issues included the absence of a comprehensive “mandatory insurance system,” incomplete insurance systems and compensation procedures, and the lack of clear differentiation between liability for clinical trial infringements and medical harm. Privacy concerns involved inadequate regulations, privacy risks associated with third-party recruitment, and data entry by clinical research coordinators. Problems related to informed consent were primarily due to inadequate awareness and capacity among investigators, a lack of oversight by ethics committees, and the absence of legal standards for broad informed consent.

**Conclusions:**

By improving the details of compensation rights, privacy protection, and informed consent in regulations, all parties involved in clinical trials can be encouraged to more effectively protect the rights and interests of subjects.

**Supplementary Information:**

The online version contains supplementary material available at 10.1186/s12910-025-01302-5.

## Background

Clinical trials have been crucial in advancing medical research and improving human health. However, participation in these trials carries inherent risks for subjects. To safeguard the rights of trial subjects, the World Medical Association introduced the Helsinki Declaration in 1964, with its seventh revision approved in Finland in 2024 [1]. The Helsinki Declaration emphasizes that while medical research aims to generate new knowledge, it must never compromise the rights of individual research subjects [2]. Therefore, prioritizing the protection of trial subjects’ rights is essential in clinical trials [3].

China became a member of the International Council for Harmonization of Technical Requirements for Pharmaceuticals for Human Use (ICH) in 2017. This development marks China’s commitment to aligning its clinical trials with international standards, thereby enhancing ethical and quality standards in the country’s clinical research. However, in terms of protecting trial subjects’ rights, China is still in the early stages of development, and ethical review institutions dedicated to safeguarding these rights are relatively nascent.

The successful conduct of clinical trials depends on effective communication and trust among trial subjects, investigators, sponsors, ethics committees, policymakers, and regulatory authorities [4]. Only through such collaborative efforts can the rights of trial subjects be more effectively safeguarded in clinical trials. In this study, we conducted a comprehensive literature review on the protection of trial subjects in Chinese clinical trials. We identified existing deficiencies in human subject protection and proposed methods for improvement. Using the Delphi expert consultation method, we administered questionnaires to experts representing clinical trial stakeholders—research institutions, ethics committees, regulatory bodies, sponsors, and third-party organizations. Our goal was to assess the current consensus among experts regarding deficiencies and potential enhancements in the protection of trial subjects in China, with the overarching objective of strengthening the protection of their rights.

This paper primarily investigates issues related to the protection of trial subjects’ rights in the areas of compensation, privacy, and informed consent within the context of clinical trials. Additionally, it explores strategies for enhancing the protection of these rights in these domains.

## Methods

### Literature search and questionnaire development

The questionnaire used in this study was developed for this study, please refer to Table S1. For this study, we conducted an extensive search of Chinese literature to gather information on issues and strategies for enhancing the protection of trial subjects’ rights. The insights derived from this literature review formed the basis for developing a survey questionnaire. We retrieved Chinese literature on the safeguarding of trial subjects’ rights in clinical trials, covering the period from 2017 to April 2022, using databases such as CNKI and Wanfang. Our search employed keywords including “subject,” “rights,” “protection,” “compensation,” “privacy,” and “informed consent” to identify and consolidate relevant information on the protection of trial subjects in Chinese clinical trials. Since the purpose of this session was to collect opinions from scholars in the field of clinical trials, all articles expressing relevant views were included. We then extracted expert views and findings from the literature and transformed these into a structured questionnaire for further investigation. The items were categorized into three areas: compensation, privacy, and informed consent. We believe these categories reflect current concerns in the field of clinical trials.

### Delphi method

This study employed a modified Delphi method, which was conducted over two rounds. Based on content identified from the literature, the study aimed to gather expert insights on issues related to protecting trial subjects’ rights in Chinese clinical trials. Additionally, it aimed to collect expert opinions on measures for enhancing the protection of these rights, particularly in the areas of compensation, privacy, and informed consent. The Delphi technique is a collaborative approach designed to establish consensus among experts in a specific field, involving a group of such experts to collectively agree on the subject matter. Following established guidelines, a team of 15–20 experts was assembled for this study [5].

Considering the practical aspects of our research, we selected experts representing clinical trial stakeholders from policy formulation, research, implementation, and oversight. The expert panel included representatives from sponsor/CRO (Contract Research Organization) entities, SMO (Site Management Organization), investigators, institutional managers, and ethics committees, amounting to a total of 16 experts. Delphi experts were purposively sampled and invited from the research team’s professional network in clinical trial management and implementation. All participants held substantial practical experience and specialized knowledge in this field. The detailed criteria for expert selection were as follows: (1) Inclusion of experts from various sectors involved in clinical trials, including regulatory authorities, institutional/organizational management, ethics committees, key investigators, sponsor representatives, and clinical research coordinators; (2) A diverse range of backgrounds within pharmaceutical-related professions; (3) Over 5 years of practical experience in the clinical trial industry; (4) Educational attainment of a bachelor’s degree or higher; (5) Familiarity with, or long-term involvement in, the field relevant to this study.

#### Scoring method

To measure the experts’ agreement with the identified issues, a 5-point Likert scale was used. The scale included five options: “strongly agree” (5 points), “agree” (4 points), “neutral” (3 points), “disagree” (2 points), and “strongly disagree” (1 point).

#### Questionnaire distribution and collection

The second-round questionnaire was designed to re-evaluate items for which consensus was not achieved during the first round. It also included additional insights from experts for reference. After two rounds of surveys, a list of items widely accepted by the expert panel was established. Experts who selected “strongly agree” or “agree” were considered to be in agreement with the respective item, with the top three agreement rates indicating issues broadly recognized by the expert group.

#### Statistical methods

Cronbach’s alpha was used to assess the internal consistency among indicators. The agreement rate, representing the combined percentage of “agree” and “strongly agree” responses, was used to evaluate the level of agreement among experts on each item. Items with the top three agreement rates were considered valid issues widely accepted by the expert group. The coefficient of variation (CV) and weighted average were applied to evaluate the alignment of expert opinions on each indicator. Specifically, CV, calculated as the standard deviation of scores divided by the mean score, indicated concentrated expert opinions and strong coordination when CV was ≤ 0.25. Additionally, the positivity index and authority coefficient were used to assess expert enthusiasm and expertise in the field. The Kendall coefficient was also used to assess the internal consistency among experts.

### Ethics approval

Approval was obtained from the Ethic Committee of Shenzhen Maternity & Child Healthcare Hospital, protocol number SFYLS[2022]030. Data were collected via an online questionnaire distributed to domain experts. The first page of the questionnaire clearly stated the study purpose, content, measures for data anonymization and confidentiality. Invited experts who, after reading this introductory information, proceeded to complete and submit the questionnaire were considered to have provided informed consent. All collected data were treated with strict confidentiality and used solely for the purposes of analysis and reporting within this study. The first page of the questionnaire will also inform the expected need to fill in the second questionnaire.

## Results

### Delphi method questionnaire items

#### Literature search results and initial questionnaire items

A total of 30 articles from 2018 to 2022 were included in the literature review. The starting point for the literature search was 2018, following the issuance of China Good Clinical Practice in 2018 and a new version of the regulations in 2020. Since the purpose of this session was to collect opinions from scholars in the field of clinical trials, all articles expressing relevant views were included. After merging similar items, the initial questionnaire consisted of 46 items related to the safeguarding of trial subjects’ rights. After deliberation and consultation with three experts, these items were further categorized into three distinct levels, based on their corresponding areas of responsibility. The items included 13 related to compensation rights, 10 related to privacy rights, and 23 related to informed consent rights. For precise classifications and detailed item information, please refer to Table S1.

The first round of the questionnaire was distributed to 16 experts in April 2022. Experts are from Guangdong(13), Hubei(2) and Fujian(1) provinces.The experts posed 12 new questions in the open-ended section; see Table S2. After sorting the data and incorporating the new questions, the results from the first round, along with the new questions for the second round, were sent to the same experts in May 2022.

#### Questionnaire reliability test

The Cronbach’s alpha coefficients for each dimension in the first round of expert consultation were all greater than 0.8, indicating excellent internal consistency of the questionnaire. For further details, please refer to Table S3 and Table S4.

#### Data reliability test

##### Expert positivity index

Before agreeing to participate in the project, experts were informed that multiple rounds of questionnaire distribution might be necessary. As a result, the expert positivity index regarding the survey outcomes reflects a high level of engagement. For detailed information, please refer to Table S5.

##### Expert authority index

Based on the criteria for expert selection, 16 experts were invited to participate in the survey. The survey outcomes showed that 7 experts (43.75%) had a strong understanding of trial subject rights protection, 2 experts (12.5%) had a relatively comprehensive understanding, and 3 experts (18.75%) had a general level of familiarity. Additionally, 2 experts (12.5%) were between relatively familiar and somewhat familiar, 1 expert (6.25%) had familiarity between general and relatively familiar, and 1 expert (6.25%) had familiarity between relatively unfamiliar and general. Notably, no expert reported being entirely unfamiliar with the subject matter. Therefore, all 16 experts were retained, as their suggestions and opinions were considered representative.

This survey study conducted statistical analyses based on the data from the original forms completed by the experts, including the “Judgment Criteria and Its Impact Quantification Table” and the “Expert Familiarity with the Issues Table.” The findings are presented in Table S6.

The table shows that the average values of expert authority levels exceed 0.8, indicating a relatively high level of expertise among the participating experts. Consequently, the accuracy of the study’s findings is notably high.

##### Expert opinion coordination coefficient

Tables S7 and S8 show that the W values for the first-round survey are 0.253 and 0.204, respectively. In both cases, the chi-square tests indicate significance (*P* < 0.01), highlighting the strong expert coordination and high reliability of the results.

### Demographics

All experts have worked in the GCP industry for 7 to 30 years, with 87.5% having 10 or more years of experience. The proportion of experts with a master’s degree or higher is over 68%. For further details, please refer to Table 1.

**Table 1:** Demographics of panel members in the Delphi technique (*N* = 16)

Basic Information	Classification	Number	Proportion
Gender	Male	5	31.25%
	Female	11	68.75%
Age	30 ~ 39	5	31.25%
	40 ~ 49	6	37.50%
	50 ~ 59	2	12.50%
	≥ 60	3	18.75%
Education	Bachelor’s degree	5	31.25%
	Master’s degree	9	56.25%
	Doctoral degree	2	12.50%
Academic Background	Clinical Medicine	7	43.75%
	Pharmacy	5	31.25%
	Clinical Medicine and Pharmacy	1	6.25%
	Nursing and others	1	6.25%
	Others	2	12.50%
Position	Regulatory Department Expert	2	12.50%
	Institutional Director/Deputy Director	1	6.25%
	Institutional Office Director	3	18.75%
	Ethics Committee Office Director/Deputy Director/Member*	6	37.50%
	PI/Sub-I	3	18.75%
	CRA/CRC	3	18.75%
	Others	2	12.50%
Years of Work in the Field (years)	7 ~ 9	2	12.50%
	10 ~ 19	9	56.25%
	20 ~ 29	4	25.00%
	≥ 30	1	6.25%

*4 individuals concurrently holding other positions

### Delphi results

Using the Delphi method, an expert panel was convened for a second round of surveys. The top three identified issues [6], along with their corresponding countermeasures related to compensation rights, privacy rights, and informed consent rights, are detailed in Tables 2, 3 and 4, followed by further discussion.

**Table 2:** Summarizes the results regarding compensation rights

Issues	Percentage of agreement	Coefficient of variation (CV)	Weighted average	Literature search or expert proposals for countermeasures	Percentage of agreement	Coefficient of variation (CV)	Weighted average
Due to the absence of specific legislation, certain courts, when dealing with cases, fail to distinguish between the liability associated with clinical trials and that related to medical damages. Instead, they apply the principles of tort liability by referring to the relevant provisions of medical damage liability.	81.25%	0.20	4.06	In Phase I, the chosen attribution principle is no-fault liability, while in Phases II to IV, the chosen attribution principle is presumed fault liability^18^.	50.00%	0.41	3.29
				It is advisable to apply the principle of no-fault liability to non-therapeutic clinical trials and the principle of presumed fault liability to therapeutic clinical trials^18^.	56.25%	0.25	3.57
While China has made initial strides in establishing a clinical trial insurance system, it does not constitute a genuine “mandatory” insurance system.	81.25%	0.20	4.05	Sponsors should offer mandatory insurance for clinical trials involving high-risk medical devices^18^.	87.50%	0.16	4.38
				Mandatory insurance should be provided by sponsors for clinical trials involving vulnerable subjects^17^.	87.50%	0.16	4.32
				Sponsors should supply mandatory insurance for drug clinical trials^18^.	75.00%	0.21	4.01
Clinical trial institutions have overly complex or protracted procedures for reimbursing compensation to human subjects.	81.25%	0.15	4.00	Clinical trial institutions should establish a relatively independent mechanism for communication or complaints regarding compensation for human subjects^43^.	68.75%	0.3	3.9
				Insurance companies and individual projects should designate dedicated personnel to assist with insurance claims for Adverse Events (AE) or Serious Adverse Events (SAE)^44^.	68.75%	0.24	4

**Table 3:** Summarizes the results regarding privacy rights

Issues	Percentage of agreement	Coefficient of variation (CV)	Weighted average	Literature search or expert proposals for countermeasures	Percentage of agreement	Coefficient of variation (CV)	Weighted average
Recruiting human subjects through third-party companies carries the risk of privacy breaches.	81.25%	0.14	3.98	Ethical committees should intensify their scrutiny of privacy risks linked to third-party recruitment.^28^	100.00%	0.15	4.43
There is a potential privacy breach risk when Clinical Research Coordinators (CRCs) use investigators’ accounts to access and review human subject information during their daily work, with no clear accountability in case of a leak.	81.25%	0.22	3.94	Set up distinct work accounts for Clinical Research Coordinators (CRCs) and restrict their access permissions.	93.75%	0.14	4.26
				Ensure the signing of confidentiality agreements.	87.50%	0.16	4.26
				Enhance the management of CRC permissions, conduct de-identification processes within the in-house systems (Clinical Trial Management Systems - CTMS), and implement automatic de-identification of electronic medical records upon access.	81.25%	0.21	4.36
The absence of comprehensive guidelines for the protection of human subject privacy is a notable issue.	75.00%	0.21	4.09	Clearly define regulations pertaining to the protection of human subject privacy in clinical trials.	100.00%	0.12	4.46
				Establish standard operating procedures for safeguarding human subject privacy and a mechanism for accountability.	93.75%	0.14	4.45

**Table 4:** Summarizes the results regarding informed consent rights

Issues	Percentage of agreement	Coefficient of variation (CV)	Weighted average	Literature search or expert proposals for countermeasures	Percentage of agreement	Coefficient of variation (CV)	Weighted average
There is a lack of legal norms governing the precise execution of comprehensive informed consent for clinical trial human subjects.	93.75%	0.16	4.26	The scope and prerequisites of comprehensive informed consent should be augmented within the pertinent legal regulations governing clinical trials.	81.25	0.11	4.29
Investigators exhibit a deficiency in awareness and competence regarding informed consent, often not affording it the requisite attention.	81.25%	0.16	4.24	Enhanced training and assessment programs for investigators on Good Clinical Practice (GCP) regulations are imperative to elevate awareness regarding the safeguarding of human subjects’ informed consent rights. ^45^	100	0.11	4.62
				Prior to the formal initiation of a trial, institutions or research departments should orchestrate standardized training sessions for investigators, encompassing the entirety of the human subject informed consent process, spanning informed consent discussions, documentation, signatures, and more.	93.75	0.14	4.28
				Departments entrusted with project implementation should enlist dedicated research physicians who focus primarily on clinical trials.	81.25	0.17	4.14
The formalization of the record format within the informed consent process does not adequately capture the essence of the informed consent procedure.	81.25%	0.19	4.09	A fortified oversight regime for the actual informed consent process, incorporating factors such as the venue, conversation content, duration, etc., is essential.	87.5	0.16	4.29
				Ethical committees ought to conduct routine or ad hoc inspections of departments to ensure that the execution of informed consent aligns with GCP and protocol stipulations.	81.25	0.15	3.99

#### Issues and countermeasures regarding compensation rights

For further details, please refer to Table 2.

#### Issues and countermeasures regarding privacy rights

For further details, please refer to Table 3.

#### Issues and countermeasures informed consent rights

For further details, please refer to Table 4.

## Discussion

China has not yet established a comprehensive insurance system (81.25%). Sponsors should be mandated to provide insurance for clinical trials involving high-risk medical devices (87.5%). Good Clinical Practice in China stipulates that the insurance and protection provided by sponsors should correspond to the associated risks [3]. However, other clinical trial regulations do not explicitly address insurance requirements. New Zealand’s clinical trial compensation guidance does not impose mandatory insurance provisions, but ethics committees have the authority to disapprove clinical trial projects that lack insurance coverage [7]. Bolland M J et al. [7] argue that sponsors should be required to obtain insurance for clinical trials, with the coverage amount based on the trial’s risks and the number of subjects. A survey conducted among research institutions and ethics committees participating in NIH-funded research projects in Africa reveals that most ethics committees believe that insurance for subject-related injuries should be the responsibility of the party benefiting from the research—the sponsor—rather than the government’s healthcare system [8]. China’s health insurance regulators also stipulate that clinical trial-related expenses should not be covered by the Chinese health insurance system. However, some scholars argue that when patients serve as research subjects and require medical treatment, they should receive prompt diagnosis and treatment according to established medical standards, in line with the “People’s Republic of China Social Insurance Law” [9]. Participation in clinical trials should not compromise or impede access to basic diagnostic and treatment services for patient-subjects, aligning with the primary purpose of establishing medical insurance funds.In China’s healthcare system, medical insurance primarily serves high-priority patients. For emergency situations requiring immediate care, insurers may provide coverage on a case-by-case basis. However, non-emergency conditions or long-term treatments for adverse events should be covered by the sponsor. Therefore, we recommend that sponsors obtain insurance for clinical trials to ensure participants receive adequate treatment. Meanwhile, medical insurance could be considered to partially cover emergency medical expenses, thereby guaranteeing timely intervention when life-threatening situations arise.

Clinical trial institutions in China face challenges due to overly complex or lengthy procedures for subject compensation reimbursement (81.25%). Like in New Zealand, China’s clinical trials lack specific guidelines regarding compensation amounts and timelines for claims [7]. Scholars such as Mackay C B et al. [10] argue that subjects often encounter delays in communication and compensation processes. Mamotte N et al. [8] advocate for the implementation of standardized and streamlined procedures to manage compensation for trial-related harm. Countries such as Russia, France, Spain, and Japan have relatively comprehensive insurance compensation standards [11], though they differ in specifics. In India, the 2014 revised Drugs and Cosmetics Rules provide detailed provisions for trial-related harm, including adverse reactions, protocol violations, investigator negligence, adverse reactions related to placebos, and clinical trial procedures. The rules also offer a formula for calculating compensation amounts. China could consider adopting and adapting these international standards to develop insurance compensation guidelines suited to its national context. Additionally, delays in the compensation process may arise from the investigator’s assessment of adverse events. Although the collection and reporting of adverse events during clinical trials in China have become more standardized with the issuance of relevant laws and regulations, there remains a lack of unified standards for determining adverse events. In September 2023, the China National Medical Products Administration released the “Technical Guidelines for the Evaluation of Adverse Event Causality in Drug Clinical Trials”, providing comprehensive guidance for investigators and sponsors in assessing adverse events. This is expected to reduce the time spent on disputes regarding adverse event determinations following harm to subjects.

A significant majority of experts (81.25%) agree that some courts, when adjudicating compensation cases related to harm suffered by clinical trial subjects, fail to differentiate between liability for clinical trial infringement and liability for medical damage. Instead, they directly apply the principles of fault liability outlined in the provisions for medical damage liability. An analysis of 12 cases involving clinical trial infringement in China revealed that more than half were determined to involve disputes related to medical damage liability [12]. Some scholars advocate for applying the principle of no-fault liability to non-therapeutic clinical trials and the principle of presumed fault liability to therapeutic clinical trials. However, only 56.25% of experts support this view. The “Civil Code of the People’s Republic of China” defines medical damage liability as follows: “If a patient is harmed during medical activities, and the medical institution or its medical staff is at fault, the medical institution shall bear compensation liability.” Regarding liability for clinical trial infringement, there is no clear definition in the regulations. This liability should pertain to harm suffered by subjects participating in clinical trials, with medical institutions or sponsors being liable for compensation. Article 1165 of the Civil Code outlines the principles of fault liability and presumed fault liability: fault liability applies when a person causes harm to others due to negligence, leading to liability for infringement; presumed fault liability applies when, according to the law, a person is presumed to be at fault and cannot prove their innocence, resulting in liability for infringement. Article 1166 details the principle of no-fault liability: if a person causes damage to the civil rights and interests of others, regardless of fault, and the law requires them to bear tort liability, it must be done in accordance with the law. In contrast, New Zealand has reached a consensus on applying the principle of no-fault liability for harm related to clinical trials [7]. Sponsors or research institutions are not required to prove fault, and subjects can receive compensation, aligning more closely with ethical principles. An example of applying the principle of fault liability is provided by the “Human Experimentation Act” in the Netherlands, where comprehensive insurance serves as strong support [13]. Another model that combines the advantages of both principles is the classification and choice solution presented by the French “Law on Bioethics,” which addresses the challenge of establishing causation for subjects [14]. The no-fault liability principle seems appropriate if discussed in terms of the protection of the rights and interests of the subjects and the timely availability of compensation.

There is a significant risk of privacy breaches (81.25%) when recruiting trial subjects through third-party companies, especially when using internet and social media platforms. This increased risk arises from the nature of online recruitment methods. Current privacy protection measures in clinical research primarily focus on informed consent, private spaces, anonymization, specimen coding, and data encoding. However, these measures are insufficient to reduce the potential risk of data theft [15]. Large data platforms, third-party recruitment platforms, and biobanks may contain identifiable information about subjects, raising privacy concerns [16, 17]. To mitigate these risks, some experts suggest limiting third-party access to biological samples and related data, and emphasize the role of ethics committees in supervision and continuous review during clinical trials [18, 19]. Ulrich et al. argue that consent for information sharing should be obtained by the subjects’ doctors rather than research assistants or volunteers, and Adarmouch et al. propose involving the subject’s primary physician in recommending trial participation, with the final decision resting with the subject [20, 21]. In China, not all clinical trial projects utilize third-party recruitment. Most researchers prefer to recruit from their own “patient pools”. By enhancing their management of patients with various conditions, research institutions can leverage their existing patient databases when undertaking clinical trials. This approach may effectively prevent privacy breaches associated with third-party recruitment while significantly reducing protocol violations or participant harm caused by insufficient understanding of participants’ medical histories through external recruitment channels.

Furthermore, there is a substantial risk (81.25%) that Clinical Research Coordinators (CRCs) may use investigators’ accounts to access subject information, potentially leading to privacy breaches without clear accountability. To address this issue, institutions should enhance CRC management by creating separate work accounts for CRCs with restricted permissions. It is essential to ensure that CRCs’ access to unrelated trial content is traceable and subject to supervision (93.75%). However, due to outdated systems in many institutions, regulating CRCs through information systems remains challenging. Adarmouch suggests that third-party data entry personnel should be subject to specific legal restrictions, such as limiting data entry to the office and obscuring identifying information. Additionally, they recommend enhancing investigator training in patient privacy protection [21]. Organizations responsible for data storage and management, such as sponsors, medical institutions, and research institutions, could explore and develop systems based on decentralized blockchain technology to ensure data integrity and prevent tampering. This approach would help ensure fair and transparent clinical trials while enhancing data security [22].

In China, the current system for protecting human subject privacy is incomplete, with privacy protection regulations being fragmented and lacking detailed rules for safeguarding subject privacy (75%). When formulating regulations and standard operating procedures for human subject privacy protection, all parties involved in clinical trials face a lack of clear operational reference standards. Wolf et al. note that privacy legislation in various countries often lags behind, proving inflexible and unable to keep pace with the continuous expansion of health data usage, rapid technological advancements, and the growing accessibility of health information [23]. To address this issue, China should develop legal policies and guiding principles specifically tailored to human subject privacy protection in clinical trials. These policies should provide clear directives and constraints for all entities involved in clinical trials, ensuring better protection of human subjects’ privacy rights [24]. Adarmouch et al. emphasize the need for robust data protection laws to regulate the collection, processing, disclosure, and utilization of personal data, as well as to protect individuals’ privacy rights [21]. They argue that interdisciplinary and inter-institutional collaboration among clinical doctors, investigators, ethics committee members, policymakers, and other stakeholders is essential for formulating effective policies [20].

The lack of adequate emphasis on informed consent by investigators, combined with their limited knowledge and skills in this area, and the demanding nature of clinical work, often results in insufficient time and effort being dedicated to clinical trials. Informed consent processes frequently focus on protecting investigators and sponsors from potential future litigation risks, but they may not effectively communicate the complexities of the clinical trial to subjects [25]. Bolland et al. argue that subjects often do not fully understand certain information in the informed consent document, leading to a failure to achieve true informed consent [7]. Despite efforts to improve subjects’ understanding of clinical trials, they frequently lack a comprehensive grasp of the potential risks, benefits, and personal data protection involved in these trials [26]. To address this issue, investigators should allocate more time for subjects to understand the contents of the informed consent document, ask questions, voice concerns, and ultimately make informed decisions about their participation in the clinical trial [4, 27]. Enhancing subjects’ comprehension of the research content and process can improve their willingness to participate and their compliance after enrollment [28]. Therefore, investigators should prioritize ensuring that subjects fully understand the informed consent document, guaranteeing that the choices they make are well-informed [29]. Departments responsible for project management may consider hiring dedicated research doctors focused primarily on clinical trials (81.25%). Additionally, institutions or research departments should provide standardized training to investigators on the entire informed consent process (93.75%). Simplifying the informed consent form to enhance readability and using multimedia video formats to obtain informed consent from subjects can standardize the process and increase investigators’ confidence in the adequacy of subjects’ informed consent [4, 25]. Enhancing public education to improve understanding of clinical trials can also encourage patients to engage in discussions. In Belgium, an organization called EUPATI is dedicated to increasing patient interest in medical innovation and drug development [27]. Moreover, investigators should pay particular attention to ensuring that elderly subjects or those with lower levels of education fully understand their participation in the project [28].

There are currently no specific legal norms regulating the precise implementation of broad informed consent for clinical trial subjects (93.75%), and existing normative documents lack sufficient legal constraints regarding the right to informed consent (81.25%). In the context of multinational pediatric clinical trials in Europe, where patient groups may be limited and dispersed, variations in national laws and ethical requirements, along with a lack of detailed guidance, necessitate substantial resources for preparing informed consent (IC) and approval documents. To address this, the European Union introduced the Pediatric Clinical Trial Ethical Guidelines in 2017. Additionally, Pirkko et al., in collaboration with several pediatric clinical research centers, published standardized guiding principles for obtaining informed consent from European pediatric trial subjects in 2022 [30]. In some cases, low-risk clinical trial projects may be eligible for exemptions from informed consent or may use simplified informed consent forms [31]. China could develop similar strategies tailored to different groups based on its unique circumstances. As regulations governing informed consent have yet to be established in China, some medical institutions currently implement electronic informed consent. During registration, patients encounter prompts related to this process and can choose whether to consent. Building on this foundation, we could use informed consent as a screening phase. Specifically, when candidates meet project eligibility criteria and are scheduled for enrollment, the registration system could then provide detailed project-specific information.

## Conclusion

The protection of human subject rights in China is still in its early stages. Issues related to compensation rights, privacy rights, and the right to informed consent stem from incomplete regulations and a lack of awareness among stakeholders about human subject rights. Challenges in securing compensation rights are primarily due to the absence of a fully enforced “mandatory insurance system,” incomplete insurance systems and procedures, and a lack of clear differentiation between liability for clinical trial infringement and liability for medical harm. Privacy rights are compromised by incomplete regulations and privacy risks associated with data entry by third-party recruiters and clinical research coordinators. Additionally, the national system for protecting human subjects’ privacy lacks comprehensiveness. The right to informed consent is at risk due to investigators’ insufficient awareness and skills in obtaining informed consent, as well as inadequate oversight by ethics committees. Improving the protection of human subject rights is a critical issue that Chinese clinical trials must address. Strengthening regulations will encourage all stakeholders in clinical trials to prioritize the protection of these rights. We hope that our study can provide valuable recommendations for regulatory strategies in Chinese clinical trials.

### Limitation

1. Expert Panel Scope: The panel comprised 16 seasoned experts in clinical trail, aligning with typical Delphi panel sizes (15–20) for in-depth consensus building. All experts met stringent selection criteria. However, variations in ethics practices across healthcare tiers and regional policies imply that perspectives from certain settings may require future inclusion.
2. Geographical Context: Recruitment focused on experts in Guangdong Province. As a hub for innovative clinical trials, Guangdong’s ethics landscape offers valuable insights for well-resourced, regulatory-advanced regions; yet, the generalizability of consensus items to resource-constrained areas (e.g., Central/Western China) or distinct sociocultural contexts warrants further validation.

## Supplementary Information

Supplementary Material 1.

## Data Availability

No datasets were generated or analysed during the current study.

## Abbreviations

SMOs: Site management organizations
CRO: Contract research organisation
SMO: Site management organization
CV: Coefficient of variation
IC: Informed consent
