# Supplementary material for: Research on issues in the protection of clinical trial human subjects in China: a Delphi study
Source: BMC Med Ethics. 2025 Nov 14;26:161. doi: 10.1186/s12910-025-01302-5 (PMC12619173; doi:10.1186/s12910-025-01302-5)
Supplement: Supplementary file 1 — Supplementary Material 1. [file 12910_2025_1302_MOESM1_ESM.doc]

**Table S1: First-round indicators and related bases - Issues in the protection of trial human subjects' rights in China (Initial Questionnaire)**

**Investigation and Research on the Problems and Countermeasures Related to the Protection of Subjects' Rights and Interests in Clinical Trials**

Dear experts:

Thank you for taking time out of your busy schedule to participate in this survey. This project mainly by reviewing the literature and comparing the regulations of ICH and other countries, summarize the experts and scholars' opinions and Suggestions on the protection of Chinese clinical trial subjects, questionnaire and using the GCP experts (regulatory authorities, sponsor, researchers, institutional managers and ethics committee) consultation, in order to promote the clear and refinement of relevant laws, regulations and guidelines of clinical trial subjects protection, further improve the regulatory system of clinical trial subjects protection in China. This questionnaire is divided into five parts, namely, expert authority and familiarity, basic information, right to obtain compensation / compensation, privacy right and informed consent. The second round of the survey will be conducted based on the consistency of the questionnaire recovery. This questionnaire is only used for academic research, and your information will be kept confidential. Please rest assured to answer it. Thank you once again for your help here!

| **First Part：Expert Authority and Familiarity**  **Please check the following according on your familiarity with the relevant field:** | | | | | | | | | | | | | | | | | | | | | | |
| --- | --- | --- | --- | --- | --- | --- | --- | --- | --- | --- | --- | --- | --- | --- | --- | --- | --- | --- | --- | --- | --- | --- |
| **Index** | | | | | **very familiar** | **familiar** | **more familiar** | | | **generally** | | | | | | **less familiar** | | | | **very unfamiliar** | | |
| **Compensation Rights** | | | | |  |  |  | | |  | | | | | |  | | | |  | | |
| **Privacy Rights** | | | | |  |  |  | | |  | | | | | |  | | | |  | | |
| **Right to Informed Consent** | | | | |  |  |  | | |  | | | | | |  | | | |  | | |
| **Please check the following according to your judgment of the relevant field:** | | | | | | | | | | | | | | | | | | | | | | |
| **Indicators and judgment basis** | | | | | | | | | **The degree of influence on your judgment** | | | | | | | | | | | | | |
|  |  |  |  |  |  |  |  |  | Large | | | | | | Medium | | | | | Small | | |
| **Compensation Rights** | | | | **(1)Theoretical analysis** | | | | |  | | | | | |  | | | | |  | | |
|  |  |  |  | **(2)Practical experience** | | | | |  | | | | | |  | | | | |  | | |
|  |  |  |  | **(3)Understanding of domestic and foreign counterparts** | | | | |  | | | | | |  | | | | |  | | |
|  |  |  |  | **(4)Intuition** | | | | |  | | | | | |  | | | | |  | | |
| **Privacy Rights** | | | | **(1)Theoretical analysis** | | | | |  | | | | | |  | | | | |  | | |
|  |  |  |  | **(2)Practical experience** | | | | |  |  |  |  |  |  |  |  |  |  |  |  |  |  |
|  |  |  |  | **(3)Understanding of domestic and foreign counterparts** | | | | |  |  |  |  |  |  |  |  |  |  |  |  |  |  |
|  |  |  |  | **(4)Intuition** | | | | |  |  |  |  |  |  |  |  |  |  |  |  |  |  |
| **Right to Informed Consent** | | | | **(1)Theoretical analysis** | | | | |  | | | | | |  | | | | |  | | |
|  |  |  |  | **(2)Practical experience** | | | | |  |  |  |  |  |  |  |  |  |  |  |  |  |  |
|  |  |  |  | **(3)Understanding of domestic and foreign counterparts** | | | | |  |  |  |  |  |  |  |  |  |  |  |  |  |  |
|  |  |  |  | **(4)Intuition** | | | | |  |  |  |  |  |  |  |  |  |  |  |  |  |  |
| **Second Part: Essential Information** | | | | | | | | | | | | | | | | | | | | | | |
| 1. Your Name：  2. Your gender：（1）Male （2）Female  3.Your age： Years old  4.Your professional background: (1) clinical medicine (2) pharmacy (3) nursing (4) public health (5) statistics (6) Biology (7) Others ：  5.Your highest degree is: ((1) bachelor's degree or below ((2) undergraduate students ((3) master's students ((4) doctoral students  6.Your role in the GCP area (multiple options): (1) Regulatory (2) Director / deputy director of the agency (3) Other managers of the Institutional Office (4) Director / Deputy Director of the Office of the Ethics Committee (5) Member of the Ethics Committee (6) PI / Sub-I (7) CRA (8) CRC  7.Your years in the GCP industry: years  8.Whether you have worked in other GCP related industries before your current position: (1)No (2)Yes, the specific name of your previous occupation For: , the number of years of work is: years  9.If you are a member of the clinical trial institution, the year of your first medical device / drug clinical trial institution qualification (or first filing) is (if the medical device and drug clinical trial institution first obtained the qualification (filing) time is different, please fill in the earliest year): year | | | | | | | | | | | | | | | | | | | | | | |
| **Third Part: Main contents of the questionnaire** | | | | | | | | | | | | | | | | | | | | | | |
| **Primary Classification** | **Secondary Classification** |  | **Existing Issues** | | | | | Strongly Disagree | | | | Disagree | | Average attitude | | | | | Agree | | Strongly Agree | |
| Compensation Rights | Regulations | 1 | Clinical trial compensation in our nation lacks clear legislation and judicial mechanisms[1]. | | | | |  | | |  | |  | | | |  | | | |  | |
|  |  | 2 | There exists a dispute regarding whether the informed consent form can establish the contractual relationship between the sponsor and the human subject[2]. | | | | |  | | |  | |  | | | | |  | | | |  |
|  |  | 3 | Laws and regulations do not explicitly define the legal relationship between investigators and human subjects participating in drug trials[2]. | | | | |  | | |  | |  | | | | |  | | | |  |
|  |  | 4 | Owing to this legislative gap, certain courts fail to differentiate between the infringement responsibility associated with clinical trials and the liability for medical damages[2]. | | | | |  | | |  | |  | | | | |  | | | |  |
|  |  | 5 | The absence of uniformity in the principles governing the attribution of infringement responsibility in drug trials poses challenges in judicial practice[2]. | | | | |  | | |  | |  | | | | |  | | | |  |
|  |  | 6 | Although China has, to some extent, established a clinical trial insurance system, it does not qualify as a genuine "mandatory" insurance system[3, 4]. | | | | |  | | |  | |  | | | | |  | | | |  |
|  | Institutions | 7 | The procedures for compensating human subjects by institutions are unduly intricate and involve excessively lengthy cycles[5]. | | | | |  | | |  | |  | | | | |  | | | |  |
|  |  | 8 | Domestic facilities frequently underestimate the potential risk that foreign companies, lacking subsidiaries or agencies in China, may assume liability for compensation without due consideration[6]. | | | | |  | | |  | |  | | | | |  | | | |  |
|  | Ethics Committees | 9 | The ethics committees of trial centers exhibit insufficient review capabilities, thereby heightening the likelihood of violations of human subjects' rights[5]. | | | | |  | | |  | |  | | | | |  | | | |  |
|  | Sponsers | 10 | Problems within the compensation clauses for human subjects in project contracts are evident in the template documents[7, 8]. | | | | |  | | |  | |  | | | | |  | | | |  |
|  |  | 11 | Human subjects are only eligible to receive compensation upon the completion of the entire clinical trial follow-up[9]. | | | | |  | | |  | |  | | | | |  | | | |  |
|  |  | 12 | Disputes concerning compensation liability for harm to human subjects frequently arise in Investigator-Initiated Trials (IIT) [10]. | | | | |  | | |  | |  | | | | |  | | | |  |
|  |  | 13 | It is often challenging for human subjects to secure compensation for mental distress[11]. | | | | |  | | |  | |  | | | | |  | | | |  |
| Privacy Rights | Third Parties | 1 | Site Management Organization (SMO) companies and Clinical Research Coordinators (CRC) often fall short in ensuring the adequate protection of human subject privacy. [12, 13] | | | | |  | | |  | |  | | | | |  | | | |  |
|  |  | 2 | The recruitment of human subjects through third-party entities introduces the risk of privacy breaches [14, 15]. | | | | |  | | |  | |  | | | | |  | | | |  |
|  | Regulations | 3 | National regulations pertaining to the protection of human subject privacy are incomplete, with various privacy protection regulations being relatively scattered [16]. | | | | |  | | |  | |  | | | | |  | | | |  |
|  | Institutions | 4 | Clinical trial institutions lack the active involvement of professionals in information confidentiality and medical engineering. [17] | | | | |  | | |  | |  | | | | |  | | | |  |
|  |  | 5 | There are inherent risks associated with storing clinical trial-related data in the cloud. [18] | | | | |  | | |  | |  | | | | |  | | | |  |
|  |  | 6 | There exists a lack of clear regulations regarding the destruction or deletion of research data. [14] | | | | |  | | |  | |  | | | | |  | | | |  |
|  |  | 7 | Institutions frequently overlook whether the enterprise that owns the Electronic Data Capture (EDC) system employed by sponsors has foreign investment [19]. | | | | |  | | |  | |  | | | | |  | | | |  |
|  | Ethics Committees | 8 | Ethics committees often provide inadequate scrutiny of human subject privacy protection. [14, 18] | | | | |  | | |  | |  | | | | |  | | | |  |
|  |  | 9 | There is a noticeable absence of professionals from information confidentiality disciplines in the ethics review process. [18, 20] | | | | |  | | |  | |  | | | | |  | | | |  |
|  | Investigators | 10 | Investigators frequently fail to adequately safeguard the privacy of human subjects during the clinical trial process. [17, 18] | | | | |  | | |  | |  | | | | |  | | | |  |
| Right to Informed Consent | Regulations | 1 | Normative documents lack legal binding force concerning informed consent for human subjects. [21] | | | | |  | | |  | |  | | | | |  | | | |  |
|  |  | 2 | There is a dearth of legal norms governing the specific implementation of broad informed consent for clinical trial participants. [22] | | | | |  | | |  | |  | | | | |  | | | |  |
|  | Ethics Committees | 3 | The review of subjects' informed consent forms is often inadequate. [23] | | | | |  | | |  | |  | | | | |  | | | |  |
|  |  | 4 | There is a deficiency in the oversight of the informed consent process. [24] | | | | |  | | |  | |  | | | | |  | | | |  |
|  |  | 5 | The entry criteria and explanations for potential trial groupings are not clearly defined. [25] | | | | |  | | |  | |  | | | | |  | | | |  |
|  |  | 6 | Procedures for addressing trial-related harm to human subjects and establishing effective channels and methods for addressing trial-related injuries are not clearly defined. [26] | | | | |  | | |  | |  | | | | |  | | | |  |
|  |  | 7 | When trials involve vulnerable populations, the reasons for conducting trials exclusively with these groups are often inadequately articulated. [27] | | | | |  | | |  | |  | | | | |  | | | |  |
|  |  | 8 | Excessive use of professional terminology makes the information less accessible. [27] | | | | |  | | |  | |  | | | | |  | | | |  |
|  | Sponsors | 9 | The expected benefits are not clearly elucidated, and alternative treatment options are not adequately explained when human subjects may not directly benefit. [27] | | | | |  | | |  | |  | | | | |  | | | |  |
|  |  | 10 | Trial procedures, including all invasive measures, and the trial's duration are not clearly outlined. [25] | | | | |  | | |  | |  | | | | |  | | | |  |
|  |  | 11 | It is not explicitly stated that if human subjects are unable to provide sufficient informed consent, consent from their legal representatives should be obtained, and, where possible, the consent of the human subject should also be sought . [27] | | | | |  | | |  | |  | | | | |  | | | |  |
|  |  | 12 | After the blind trial is unblinded, human subjects are not informed about their trial group and medication conditions. [22] | | | | |  | | |  | |  | | | | |  | | | |  |
|  | Investigators | 13 | Human subjects are not informed that their participation in the trial is voluntary, and they have the right to decline participation or withdraw at any time without affecting their medical treatment or rights. [27] | | | | |  | | |  | |  | | | | |  | | | |  |
|  |  | 14 | Research doctors often lack sufficient time for comprehensive communication with human subjects, which may impede full understanding. [25] | | | | |  | | |  | |  | | | | |  | | | |  |
|  |  | 15 | Inappropriate reception of human subjects in unsuitable locations by research doctors can hinder comprehension. [23, 25] | | | | |  | | |  | |  | | | | |  | | | |  |
|  |  | 16 | Failure to promptly inform human subjects and obtain their informed consent after updating the informed consent document is common. [23] | | | | |  | | |  | |  | | | | |  | | | |  |
|  |  | 17 | The formalization of the documentation process often fails to accurately reflect the informed consent process. [23] | | | | |  | | |  | |  | | | | |  | | | |  |
|  |  | 18 | There is a risk of excessive inducement or undue influence. [25] | | | | |  | | |  | |  | | | | |  | | | |  |
|  |  | 19 | Failure to adequately consider the informed consent of the human subject, resulting in an unreasonable signing of consent by the legal representative. [27] | | | | |  | | |  | |  | | | | |  | | | |  |
|  |  | 20 | Due to the demanding clinical workload, research doctors sometimes permit unauthorized personnel, such as residents or fellows, to substitute for them in obtaining informed consent. [23, 26] | | | | |  | | |  | |  | | | | |  | | | |  |
|  |  | 21 | Having a research nurse authorized by the Principal Investigator (PI) obtain a human subject's informed consent and sign the informed consent form is considered inappropriate. [19] | | | | |  | | |  | |  | | | | |  | | | |  |
|  |  | 22 | Signing of consent forms sometimes occurs after the screening process. [27] | | | | |  | | |  | |  | | | | |  | | | |  |
|  |  | 23 | The distinction between the right to informed consent in clinical trials and the right to informed consent in diagnostic and therapeutic activities is not consistently emphasized. [22] | | | | |  | | |  | |  | | | | |  | | | |  |

**References:**

1. Bangyu Z, Yuping L, Ling Z, Ying X, Bin Z. Enlightenment of Japanese Clinical Trials Act to China. Chinese Journal of New Drugs and Clinical Remedies. 2021;40:195-200.

2. Yixi L. An Analysis on Dispute Between Ran, Li and George Company etcover Liability of Clinical Trial for Drug. 2020;49.

3. Qingsi Z, Zhongyuan X. Status Quo of Clinical Trials Insurance Practice at Home and Abroad. Medicine & Philosophy. 2020;41:56-59+80.

4. Peng H. Study on the Classification Establishment of Imputation Principle of Tort Liability of Clinical Drug Trial. Medicine & Philosophy. 2021;42:71-75+79.

5. Shengya Z, Dan L, Jiyin Z. Difficulties and Countermeasures of Compensation/Indemnification for Clinical Research Subjects. Chinese Medical Ethics. 2018;31:1368-71+76.

6. Shuang M. One case of clinical trial subjects damage compensation. The Chinese Journal of Clinical Pharmacology. 2018;34:181-83.

7. Yuran C, Guoying C, Jing Z, Cuiyun W, Huizhong Z, Ying M. Research on problems and countermeasures of the management of clinical trials' contracts in medical institutions. Chinese Journal of New Drugs. 2019;28:2997-3000.

8. Wenjing Z, Niya L, Ping H. Common problems and countermeasures of drug clinical trial contract management. Chinese Journal of New Drugs and Clinical Remedies. 2019;38:467-71.

9. E6(R2) I. Integrated Addendum to Good Clinical Practice (GCP). 2016.

10. Ye C, Liying Q, Hui J, Shuangzhen C, Can L, Ting L, et al. Thoughts and recommendations arising from a review of "subcenter contracts" for clinical research initiated by investigators. Chinese Journal of New Drugs. 2021;30:2288-94.

11. Sijia W. Research on the tort law of drug clinicaltrial in China. 2021;48.

12. Zhifang Z, Yikai F, Yashu Y, Feng Y. Risk analysis and mitigation in clinical trials conducted by contract research organization. Chinese Journal of Clinical Pharmacy. 2021;30:418-23.

13. Ting W, Zhihua Z, Fangmei L, Fangjie A, Sifan L. Supervision Mode of Drug Clinical Trial Institutions on the Performance of the Clinical Research Coordinator. Journal of Pediatric Pharmacy. 2020;26:52-55.

14. Yingshuo H, Zilong Z, Xiaofang W, Yanhong K, Ruihua D. Ethical Issues and Research Progress in Privacy Protection of Drug Clinical Trial Subjects. Chinese Medical Ethics. 2020;33:1046-52.

15. Wei C, Danping W. Ethical review of subject recruitment by third-party in clinical trials. Chinese Journal of New Drugs and Clinical Remedies. 2020;39:664-66.

16. Chao C, Ming Y, Xue L, Xiaoyun C. The Present Situation and Latest Progress of Subject Privacy Protection in Real World Study. Medicine & Philosophy. 2021;42:1-5+10.

17. Haigin L, Jing L, Minjuan Y, Yewen S, Boyan C, Yani F, et al. Ethical Thoughts on Patient Privacy Exposure under the Background of Health Big Data. Chinese Medical Ethics. 2019;32:1283-87.

18. Xianchen L, Mei K, Yan Q, Huichen H, wengian G, Biyun Q, et al. Exploring on Privacy Protection of Subjects in Investigator-Initiated Clinical Studies. Chinese Medical Ethics. 2020;33:1459-62.

19. Zhongqi Y, Minghuang H. Drug clinical trial practice and consensus. 2020.

20. Association GP. Consensus of Expert on Subject Privacy Protection of Drug Clinical Trial in Guangdong(Version 2020). Pharmacy Today. 2020;1-17.

21. Chenchen B. Study on the protection system of the rights and interests of thehuman subjects of clinical trials in Japan. 2020;36.

22. Ling N. Legal interpretation and improvement of informed consent in drug clinical trials. . 2021;7.

23. Jiangchuan X, Wei G, Linli X, Minghong L, Xinmei P, Liva C, et al. The Common Problems and Countermeasures in the Process of Informed Consent of Drug Clinical Trials. Chinese Medical Ethics. 2021;34:835-38.

24. Wenjing Z. Study on the protection of informed consent of subjects in multicenter drug clinical trials in China. . 2021;51.

25. Xiaomin X, Jiping Z, Rui D, Xiaoxia W, Jin G. Several thoughts on the question of informed consent given in clinical trials. Electronic Journal of Clinical Medical Literature. 2019;6:188-89+91.

26. Meixia W, Yiting L. Changes of Informed Consent Policy of Drug Clinical Trials in china:The Comparison between Gcp 2003 Version and 2020 Version. Medicine & Philosophy. 2020;41:12-19.

27. Liyan L. Study on Protection of Subjects’ Informed Consent Right in Clinical Trail of Medical Device. 2019;68.

Table S2: Additional items from experts in the first-round expert survey questionnaire

| **Primary Classification** | **Secondary Classification** | **Existing Issues** |
| --- | --- | --- |
| Compensation Rights | Regulations | The legal foundation for compensation and reimbursement standards remains unclear. |
|  | Sponsors | Sponsors impose numerous limitations on the scope of compensation for trial-related injuries, thereby reducing their own liability risks. |
|  |  | Following an injury, human subjects are required to cover upfront medical expenses and navigate the insurance claims process, which hinders their ability to receive timely and appropriate treatment. |
|  | Human Subjects | Human subjects often lack awareness or initiative to seek compensation and are uncertain about whether the trial has procured insurance coverage. |
| Privacy | Third Parties | The overlapping responsibilities between Clinical Research Coordinators (CRC) and Clinical Research Associates (CRA) heighten the risk of privacy breaches. |
|  | Regulations | Detailed regulations for safeguarding human subject privacy are lacking. |
|  | Ethics Committees | Ethical concerns related to compromising human subject privacy when using their data across multiple trial projects or retrospective research endeavors are challenging to identify. |
|  | Investigators | Loose management of raw data or source data containing identifiable human subject information makes it susceptible to unauthorized access. |
|  | Human Subjects | Human subjects typically possess limited awareness of privacy protection. |
| Right to Informed Consent | Investigators | The delegation of the informed consent process to CRCs, rather than having it conducted by the investigator, is observed. |
|  |  | Investigators often lack sufficient awareness and emphasis on the informed consent process. |
|  | Sponsors | Trial sponsors may intervene in human subjects' access to information to ensure desired predictive outcomes. |
|  | Human Subjects | Consequently, human subjects often lack a correct and comprehensive understanding of clinical trials, which impedes their ability to fully comprehend the information provided during the informed conversation and consent process. |

**Table S3: Reliability Statistics (Existing Issues)**

| **Dimensions** | **Cronbach's alpha** | **Number of items** |
| --- | --- | --- |
| Right to compensation | 0.833 | 12 |
| Privacy | 0.885 | 11 |
| Right to informed consent | 0.969 | 24 |

**Table S4: Reliability Statistics (Countermeasures)**

| **Dimensions** | **Cronbach's alpha** | **Number of items** |
| --- | --- | --- |
| Right to compensation | 0.918 | 24 |
| Privacy | 0.928 | 11 |
| Right to informed consent | 0.911 | 12 |

**Table S5: Positivity Index of Consulted Experts**

|  | **Distribution of expert questionnaires** | **Collection of expert questionnaires** | **Collection rate** |
| --- | --- | --- | --- |
| 1^st^ Round | 16 | 16 | 100% |
| 2^nd^ Round | 16 | 16 | 100% |

**Table S6: Overview of Expert Authority Coefficient Results**

|  | **Judgment coefficient** | **Familiarity coefficient** | **Authority coefficient** |
| --- | --- | --- | --- |
| Right to compensation | 0.93 | 0.81 | 0.87 |
| Privacy | 0.92 | 0.86 | 0.90 |
| Right to informed consent | 0.96 | 0.86 | 0.91 |

Table S7: Consistency Test Statistics for Indicators (Existing Issues)

| **Rounds** | **Sample size** | **Chi-square value** | **Kendall's concordance coefficient (W)** | **Degrees of freedom** | ***P*** |
| --- | --- | --- | --- | --- | --- |
| 1^st^ Round | 16 | 186.24 | 0.253 | 46 | <0.001 |
| 2^nd^ Round | 16 | 236.15 | 0.250 | 59 | <0.001 |

Table S8: Consistency Test Statistics for Indicators (Proposed Countermeasures)

| **Rounds** | **Sample size** | **Chi-square value** | **Kendall's concordance coefficient (W)** | **Degrees of freedom** | ***P*** |
| --- | --- | --- | --- | --- | --- |
| 1^st^ Round | 16 | 150.01 | 0.204 | 46 | <0.001 |
| 2^nd^ Round | 16 | 176.42 | 0.190 | 58 | <0.001 |
